# Recent advances in natural compounds inducing non-apoptotic cell death for anticancer drug resistance

**DOI:** 10.20517/cdr.2023.78

**Published:** 2023-10-19

**Authors:** Jia-Wen Chen, Sibao Chen, Guo-Qing Chen

**Affiliations:** ^1^Institute of Medicinal Plant Development, Chinese Academy of Medical Sciences and Peking Union Medical College, Beijing 100193, China.; ^2^State Key Laboratory of Chinese Medicine and Molecular Pharmacology (Incubation), The Hong Kong Polytechnic University Shenzhen Research Institute, Shenzhen 518057, Guangdong, China.; ^3^Department of Food Science and Nutrition, The Hong Kong Polytechnic University, Hung Hom, Hong Kong 999077, China.; ^4^Research Centre for Chinese Medicine Innovation, The Hong Kong Polytechnic University, Hung Hom, Hong Kong 999077, China.

**Keywords:** Drug resistance, cancer therapy, natural compound, non-apoptotic cell death

## Abstract

The induction of cell death is recognized as a potent strategy for cancer treatment. Apoptosis is an extensively studied form of cell death, and multiple anticancer drugs exert their therapeutic effects by inducing it. Nonetheless, apoptosis evasion is a hallmark of cancer, rendering cancer cells resistant to chemotherapy drugs. Consequently, there is a growing interest in exploring novel non-apoptotic forms of cell death, such as ferroptosis, necroptosis, pyroptosis, and paraptosis. Natural compounds with anticancer properties have garnered significant attention due to their advantages, including a reduced risk of drug resistance. Over the past two decades, numerous natural compounds have been discovered to exert anticancer and anti-resistance effects by triggering these four non-apoptotic cell death mechanisms. This review primarily focuses on these four non-apoptotic cell death mechanisms and their recent advancements in overcoming drug resistance in cancer treatment. Meanwhile, it highlights the role of natural compounds in effectively addressing cancer drug resistance through the induction of these forms of non-apoptotic cell death.

## INTRODUCTION

Cancer, as a major global public health concern, poses a severe threat to people’s well-being. In 2018, the World Health Organization reported that cancer ranked the second leading cause of death globally, resulting in approximately 9.6 million annual fatalities^[1]^. Cell death is a fundamental process crucial for human health, playing a pivotal role in regulating cell division, facilitating organ development, and upholding tissue homeostasis^[2]^. Nevertheless, the disruption and avoidance of cell death mechanisms facilitate malignant cell transformation and advance tumorigenesis^[3]^. From a therapeutic perspective, conventional cancer treatments, such as chemotherapy and radiation, achieve their anticancer effects by inducing cell death^[2]^.

Over the span of several decades, there has been a growing comprehension of the diverse mechanisms governing cell death. This process can be classified into two primary categories: programmed cell death (PCD), which is genetically controlled, and unprogrammed cell death, which represents a passive response to both biotic and abiotic stress. Among the various forms of PCD, apoptosis has emerged as one of the earliest and most extensively explored pathways. For a significant duration, the development of anticancer drugs targeting apoptosis has been a focal point of research. Numerous drugs, such as cisplatin, oxaliplatin, and pirarubicin, have showcased their ability to elicit anticancer effects through this mechanism^[4,5]^. However, recent studies have increasingly validated that cancer cells possess the capability to elude apoptosis through a range of mechanisms, including the overexpression of apoptosis-inhibiting proteins, the inhibition of apoptosis-inducing factors, and the activation of survival signaling pathways^[6]^. This evasion of apoptosis directly contributes to chemoresistance, a phenomenon intricately associated with alterations in various aspects of cancer, encompassing angiogenesis, the tumor microenvironment, and oxidative stress^[7-10]^. Ultimately, this leads to the failure of cancer treatment. To improve the efficacy of cancer treatment, the exploration of non-apoptotic cell death mechanisms has gradually emerged as a prominent priority. Over the last two decades, researchers have sequentially unveiled and extensively investigated novel different cell death mechanisms, including ferroptosis, necroptosis, pyroptosis, and paraptosis. These unique mechanisms, each with its distinct regulators and pathways, hold the potential to be activated within apoptosis-resistant cancer cells, offering novel strategies for the treatment of cancer and overcoming cancer drug resistance.

Throughout history, natural compounds derived from plants, animals, microorganisms, and minerals have consistently served as a valuable source for drug discovery^[11,12]^. Unlike synthetic compounds, these substances are not artificially created and can be categorized into various forms, such as alkaloids, flavonoids, and terpenes, due to their diverse chemical structures^[13]^. Many of these natural compounds exhibit significant potential in the realm of cancer treatment. In fact, certain natural compounds, such as paclitaxel (PTX), camptothecin, and vincristine, have already gained widespread acceptance as chemotherapeutic drugs in clinical practice^[14,15]^. Moreover, an interesting aspect is that numerous natural compounds have been found to induce various non-apoptotic cell death pathways when administered to resistant cancer cells that evade apoptosis.

This review focuses on ferroptosis, necroptosis, pyroptosis, and paraptosis, providing a comprehensive overview of the latest research advancements in these mechanisms within the framework of cancer. We place specific emphasis on their relevance in the context of combatting cancer drug resistance. Additionally, this review compiles information on natural compounds with the capacity to induce these four modes of cell death in the context of addressing cancer resistance over the past two decades.

## FERROPTOSIS

### Overview of ferroptosis

Ferroptosis, a recently discovered form of PCD that is iron-dependent, was first proposed in 2012^[16]^. Morphologically, ferroptotic cells exhibit intact nuclei without chromatin condensation. However, their mitochondria undergo significant changes, including reduced size, increased membrane density, reduced cristae, and outer membrane rupture^[17]^. Interestingly, the discovery of ferroptosis inducers was earlier than its naming. Yang *et al.* discovered some new compounds as early as 2003 and 2008, including Erastin, RSL3, and RSL5, which induce cell death through a mechanism distinct from apoptosis^[18]^. Erastin reduces cysteine uptake by inhibiting cysteine/glutamate transporter receptor, known as System X_c_^-^, resulting in a decrease in glutathione (GSH) synthesis and an increase in iron-dependent lipid peroxidation (LPO), which finally leads cells to ferroptosis^[19]^. Differently, RSL3 directly inhibits the activity of Glutathione Peroxidase 4 (GPX4), a GSH-utilizing enzyme that prevents the accumulation of toxic lipid hydroperoxide, thereby inducing ferroptosis^[20]^. Additionally, Erastin also indirectly triggers ferroptosis through the inactivation of GPX4 via the inhibition of GSH synthesis^[21]^.

It is known that LPO and intracellular iron accumulation play pivotal roles in triggering ferroptosis. Polyunsaturated fatty acids (PUFAs) are the most susceptible lipids to peroxidation during ferroptosis. The regulation of PUFA synthesis may be influenced by enzymes such as Long-chain acyl-CoA synthetases (ACSLs) and lyso-phosphatidylcholine acyltransferase-3 (LPCAT3). Additionally, arachidonate lipoxygenases (ALOXs) and cytochrome p450s (POR) can directly or indirectly modulate PUFA peroxidation, resulting in ferroptosis^[22]^. Additionally, iron metabolism is crucial for ferroptosis. As Fe^2+^ is released from the labile iron pool (LIP) into the cytoplasm, excess Fe^2+^ oxidizes PUFAs to hydroxyl radicals and leads to ferroptosis.

**Figure 1:**
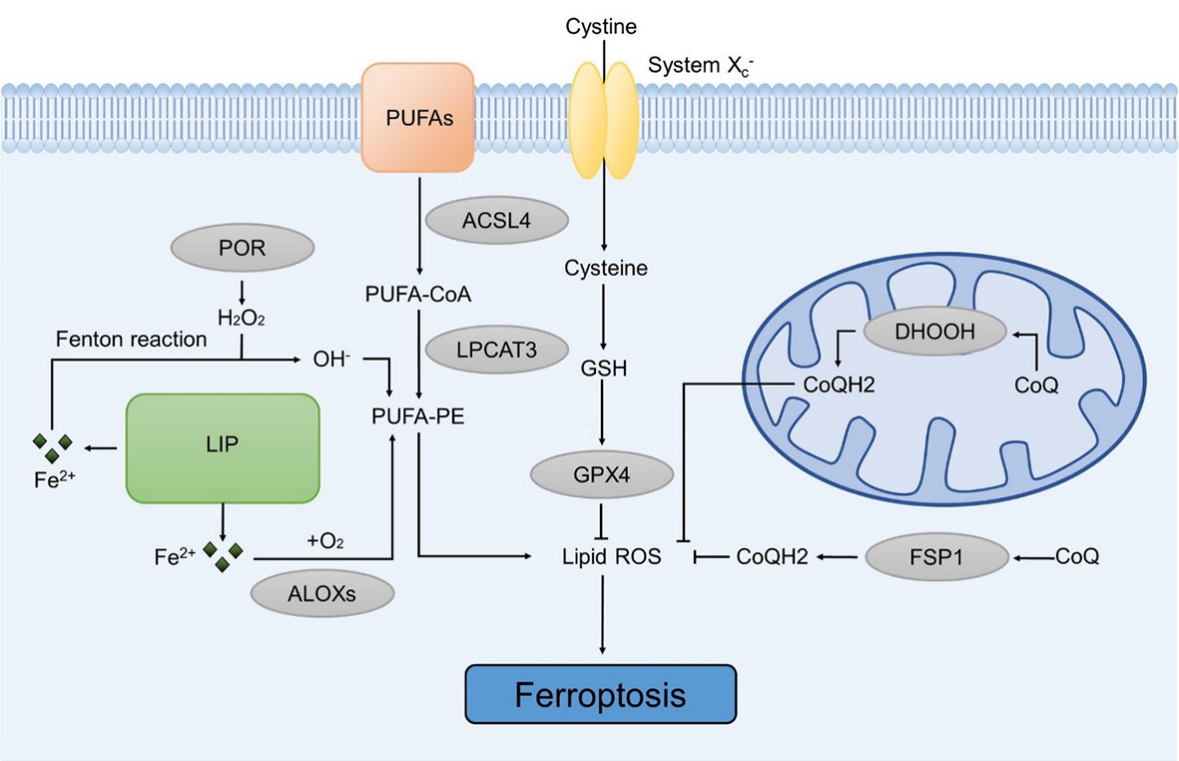
The mechanism of ferroptosis. Ferroptosis can be initiated by an increase in intracellular iron levels and the accumulation of iron-dependent lipid peroxidation. It can be induced through the SystemX_c_^-^/GSH/GPX4 axis, the DHODH/CoQ10 axis, and the FSP1/CoQ10 axis. ACSL: Acyl-CoA synthetase; ALOXs: arachidonate lipoxygenases; CoQ: ubiquinone; FSP1: ferroptosis suppressor protein 1; GPX4: glutathione peroxidase 4; GSH: glutathione; LPCAT3: lyso-phosphatidylcholine acyltransferase-3; LIP: labile iron pool; POR: cytochrome p450s; PUFA: polyunsaturated fatty acid.

The mitochondria are responsible for cellular metabolism and also play an important role in the regulation of ferroptosis^[23]^. Within the mitochondria, dihydroorotate dehydrogenase (DHODH) reduces ubiquinone (CoQ) to ubiquinol (CoQH2), serving as a radical-trapping antioxidant with anti-ferroptosis activity. DHODH plays a significant role in mediating ferroptosis defense independent of the GSH pathway^[24]^. Similarly, the plasma enzyme ferroptosis suppressor protein 1 (FSP1) inhibits lipid hydroperoxides by reducing ubiquinone to ubiquinol, operating in parallel with GPX4 to counteract ferroptosis^[25]^. Obviously, the mechanisms of ferroptosis are complex and being explored constantly [Figure 1].

### Ferroptosis pathways for chemotherapy resistance in cancer

In recent years, ferroptosis has been a hot topic in cancer development, treatment and cancer drug resistance. Several signaling pathways have been found to participate in promoting cancer development and drug resistance through the inhibition of ferroptosis. With multiple functions for proliferation, metastasis, and differentiation of cancer cells, the phosphatidylinositol 3-kinase (PI3K)/AKT/mammalian target of the rapamycin (mTOR) signaling pathway is responsible for ferroptosis prevention via sterol regulatory element-binding protein 1 (SREBP1)/stearoyl-CoA desaturase-1 (SCD1)-mediated adipogenesis^[26]^. Hippo pathway activity is also responsible for cell growth and proliferation. Activating the Hippo pathway can suppress downstream YAP, leading cancer cells resistant to ferroptosis by downregulating acyl-CoA synthetase long-chain family member 4 (ACSL4) and TCP friendly rate control (TFRC)^[27]^. Since RAS may regulate some processes to escape ferroptosis, RAS-mutated cells are always susceptible to ferroptosis. High-mobility group box 1 (HMGB1), a leukemia pathogenic gene, inhibits LPO via the RAS/MAP kinase (MAPK) pathway, promoting ferroptosis resistance^[28]^.

Additionally, several other pathways, including nuclear factor erythroid 2-related factor 2 (Nrf2), p53, and hypoxia-inducible factor (HIF), are also involved in chemotherapeutic resistance through ferroptosis regulation. Recent research highlights the significant impact of the Nrf2 signaling pathway on both organ protection and resistance to cisplatin (DDP) across various cancer types^[29]^. For instance, in non-small cell lung cancer, Erastin and Sorafenib, either alone or in combination, induce ferroptosis by inhibiting the Nrf2/SLC7A11 (also known as xCT) pathway, thereby overcoming DDP resistance^[30]^. The p53 gene, extensively studied in cancer research, influences metabolic pathways related to ferroptosis, such as enhancing ferroptosis by down-regulating xCT expression^[31]^. According to this mechanism, Flubendazole and 5-fluorouracil (5-FU) demonstrate synergistic effects in treating castration-resistant prostate cancer^[32]^. HIF serves a dual role in the regulation of ferroptosis in cancer cells. Lowering HIF-α levels has been found to increase LPO and enhance ferroptosis in clear cell renal cell carcinoma^[33]^. Conversely, in temozolomide-resistant glioblastoma, activating HIF-1α and HIF-2α can induce ferroptosis, with HIF-2α possibly promoting LPO as the primary mechanism^[34]^.

### Natural compounds inducing ferroptosis for cancer treatment

Many natural compounds have been found to induce ferroptosis via single or combinational therapies, offering opportunities for cancer treatment and drug resistance [Table 1]. Here, we summarize those natural compounds that reverse drug resistance by inducing ferroptosis. Most of these compounds can synergize with chemotherapeutic drugs by regulating ferroptosis-related proteins and genes. Recent studies have revealed that sorafenib can induce ferroptosis in various types of cancer^[35]^. However, sorafenib-induced ferroptosis can be suppressed, specifically through the activation of the PI3K/Akt signaling pathway or the NRF2/GPX4 axis^[36,37]^. To overcome this challenge, drug combinations have proven to be effective strategies. Dihydroartemisinin, a derivative of artemisinin, shares similar mechanisms with sorafenib regarding ferroptosis-related proteins, such as GPX4, and has a stronger effect in liver cancer cells when combined with sorafenib^[38]^. Ursolic acid, a pentacyclic triterpene compound, also exhibits synergistic anticancer effects with sorafenib by inhibiting xCT in many cancers^[39]^. While DDP is considered a front-line chemotherapy drug for various cancers, including ovarian cancer, its resistance poses a significant impediment to achieving effective treatment outcomes^[40]^. Inhibition of apoptosis plays a significant role in contributing to DDP resistance, whereas the induction of novel forms of cell death, such as ferroptosis, has been shown to effectively kill DDP-resistant cancer cells that evade apoptosis^[41]^. The combination of DDP with Shikonin, a hydroxy-1,4-naphthoquinone isolated from *Lithospermum erythrorhizon* Sieb. et Zucc.(Boraginaceae), promotes Fe^2+^ accumulation by upregulating heme oxygenase 1 (HMOX1), initiating ferroptosis in DDP-resistant ovarian cancer cells^[42]^. Piperlongumine, an alkaloid derived from long pepper (*Piper longum* L.), exhibits anticancer activity in lung cancer cells by targeting the glutathione regeneration enzyme, thioredoxin reductase 1 (TXNRD1). Although it does not induce ferroptosis, it can significantly enhance erastin-induced LPO^[43]^.

**Table 1:** Natural compounds for anticancer drug resistance by inducing ferroptosis

**Compounds**	**Origin**	**Structure**	**Cancer**	**Anti-drug resistant effects**	**Refs**
Artesunate	Artemisia annua L.	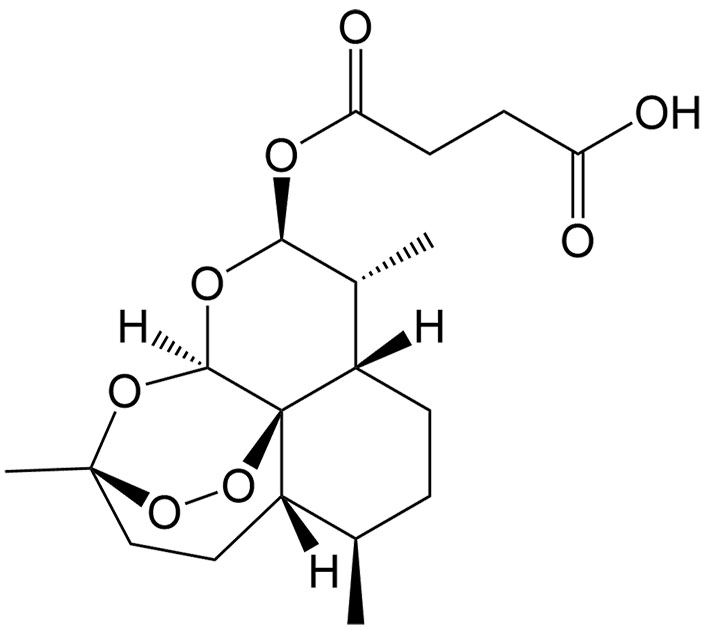	Renal cell carcinoma	Increasing cytotoxicity in sunitinib-resistant renal cell carcinoma by triggering ferroptosis, increasing ROS generation, and decreasing metabolism.	[50]
Chrysin	Oroxylum indicum (L.) Kurz	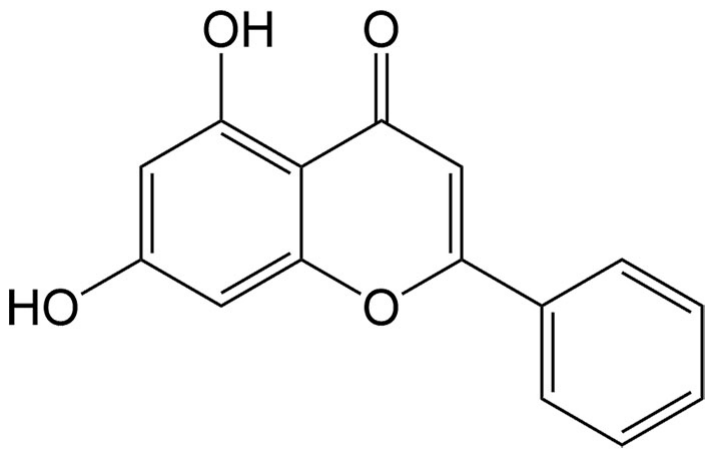	Pancreatic cancer	Inhibiting CBR1 activity in gemcitabine-resistant pancreatic cancer to trigger ferroptosis through ROS accumulation.	[48]
Dihydroartemisinin	Artemisia annua L.	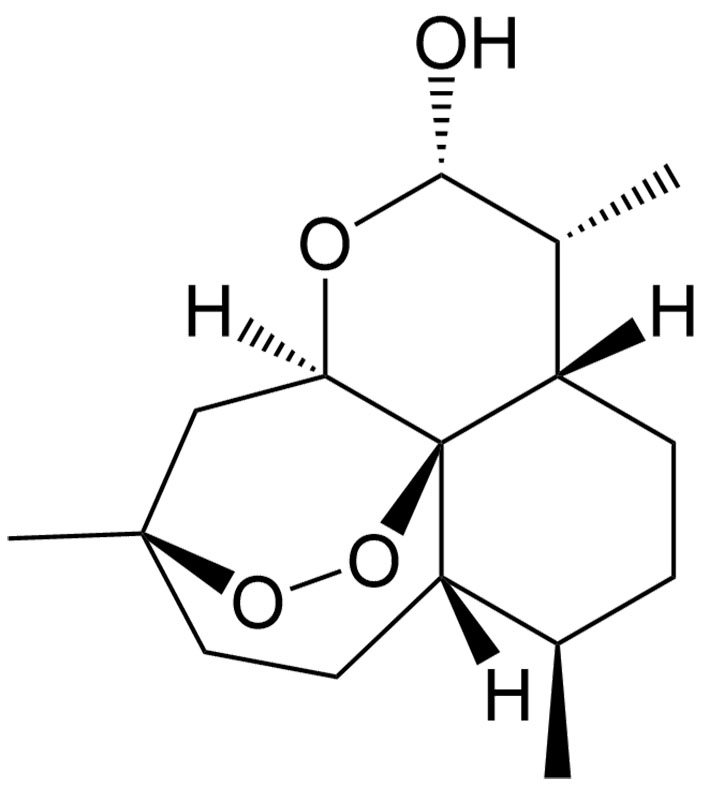	Hepatocellular carcinoma	Synergizing with sorafenib to induce ferroptosis by increasing the levels of L-ROS, LIP, and MDA and decreasing the level of GSH.	[38,127-129]
Ginkgetin	Ginkgo biloba L.	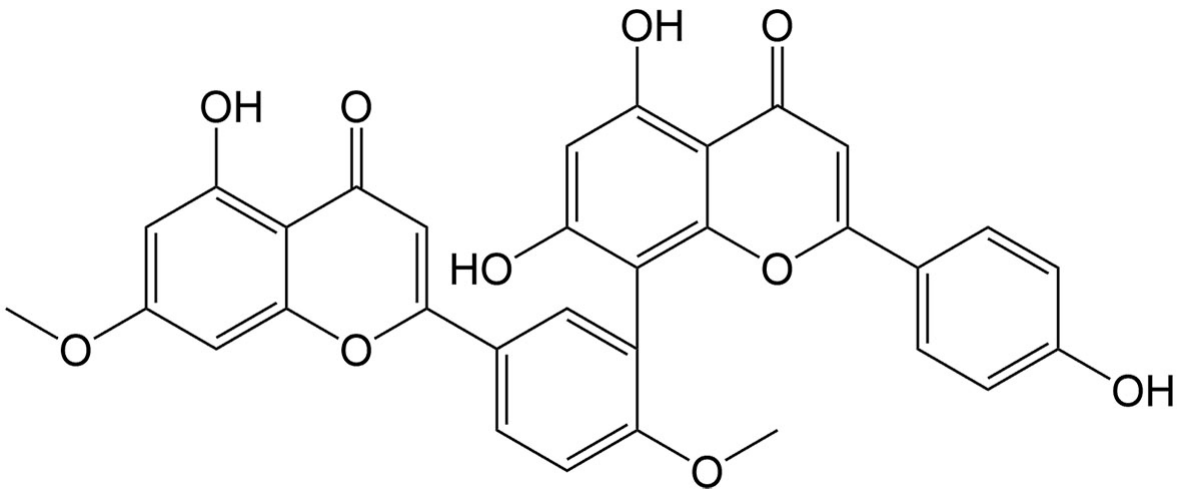	Non-small cell lung cancer	Synergizing with DDP to induce ferroptosis by increasing ROS formation, decreasing the expression of xCT and GPX4, and inactivating the Nrf2/HO-1 axis.	[46]
Kayadiol	*Torreya nucifera* Sieb. et Zucc. (Taxaceae)	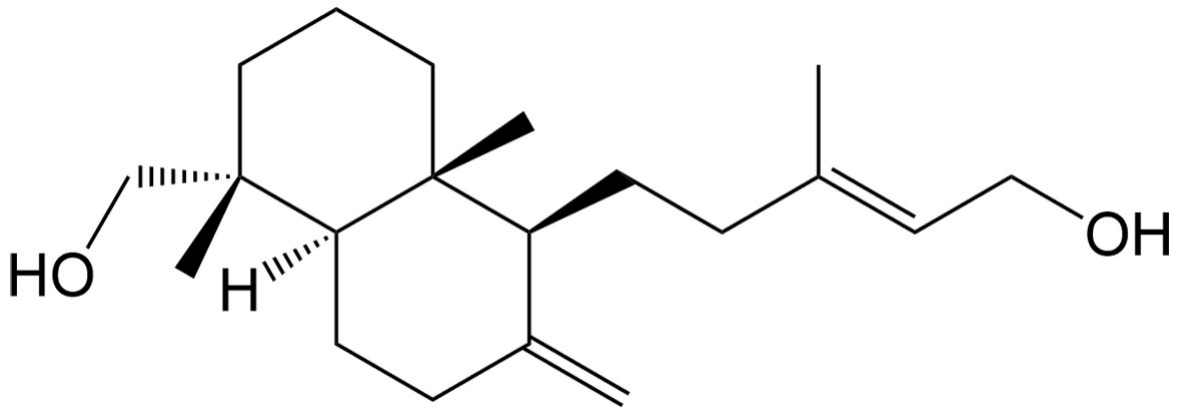	Extranodal natural killer/T cell lymphoma	Inducing p53-mediated ferroptosis through the xCT/GPX4 axis and exhibiting synergistic effects when combined with L-asparaginase and DDP.	[49]
Piperlongumine	*Piper longum* L.	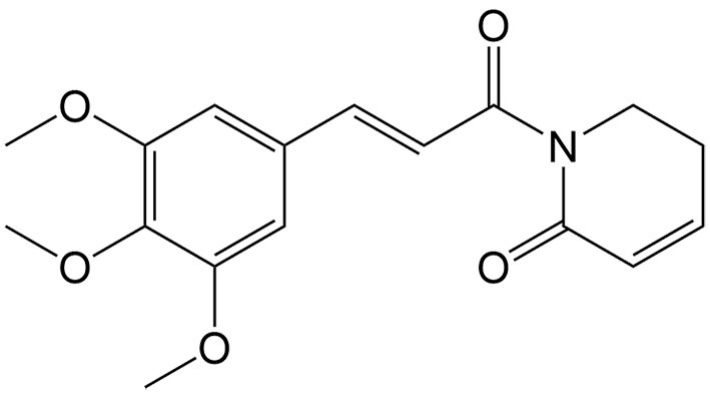	Pancreatic cancer	Enhancing the antitumor effects of erastin by inducing ROS generation, GSH depletion and inhibiting TXNRD activity.	[43,130]
Shikonin	*Lithospermum erythrorhizon* Sieb. et Zucc.	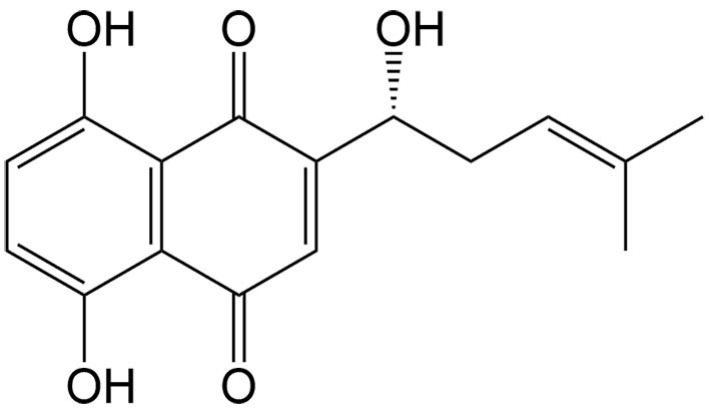	Ovarian cancer	Synergizing with DDP to induce ferroptosis through upregulation of HMOX1 and increased levels of ROS, LPO, and Fe^2+^.	[42]
Tiliroside	*Tribulus terrestris* L.	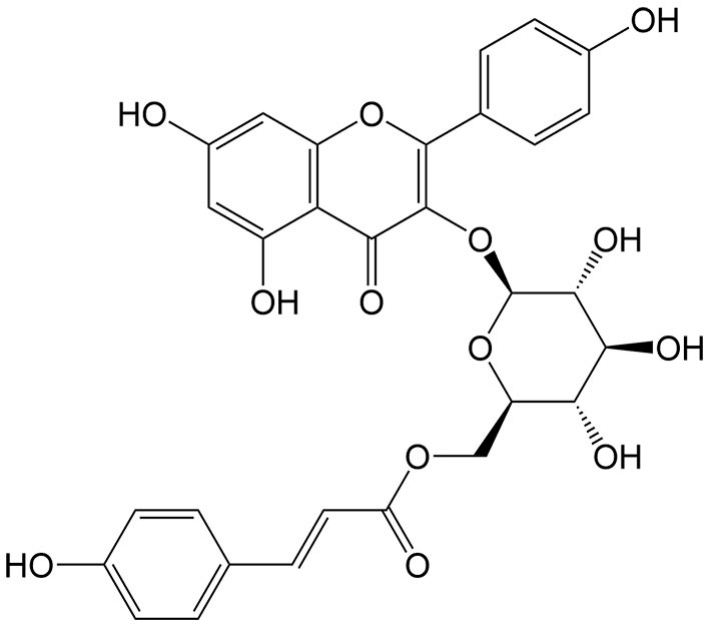	Hepatocellular carcinoma	Enhancing the antitumor effects of sorafenib by inducing ferroptosis via targeting TBK1 to promote Keap1-mediated Nrf2 ubiquitination and degradation.	[45]
Ursolic acid	*Ligustrum lucidum* W. T. Aiton	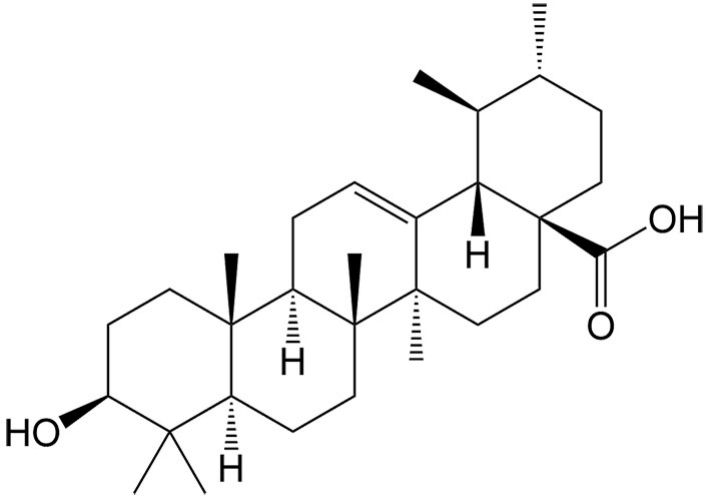	Colon cancer, gastric cancer, prostate cancer	Enhancing the antitumor effects of sorafenib by inducing xCT-dependent ferroptosis.	[39]
Withaferin A	*Withania somnifera* (L.) Dunal	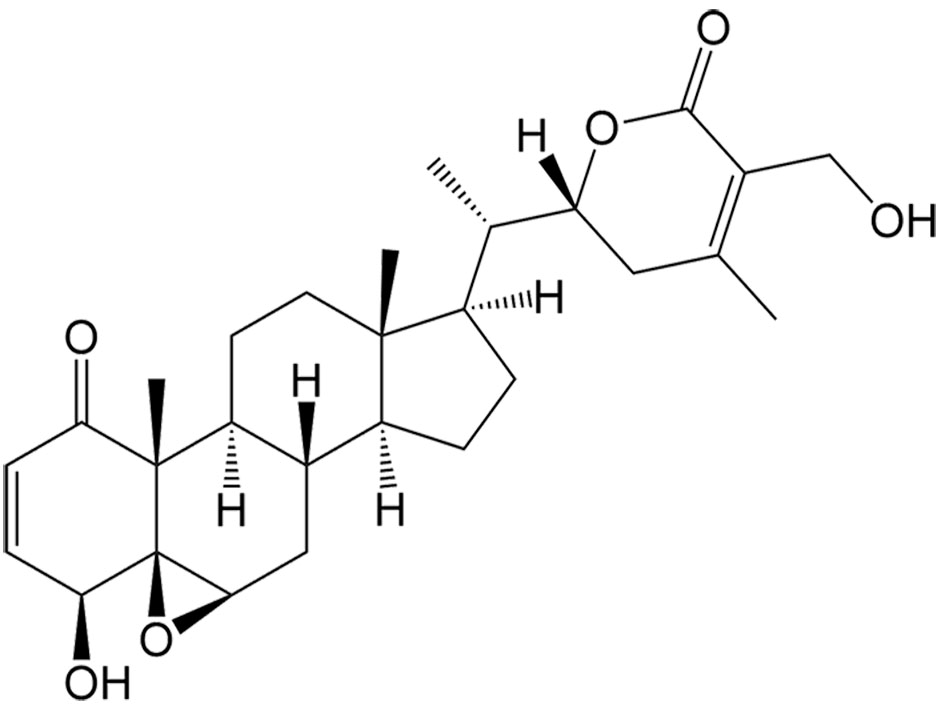	Hepatocellular carcinoma	Enhancing the antitumor effects of sorafenib in sorafenib-resistant hepatocellular carcinoma cells by regulating Keap1/Nrf2-associated ferroptosis and EMT.	[44]

CBR1: Carbonyl reductase 1; DDP: cisplatin; EMT: mesenchymal transition; GPX4: glutathione peroxidase 4; GSH: glutathione; HMOX1: heme oxygenase 1; LIP: labile iron pool; LPO: lipid peroxidation; L-ROS: lipid reactive oxygen species; MDA: malondialdehyde; TBK1: tank-binding kinase; TXNRD: thioredoxin reductase.

In addition to ferroptosis-related genes and proteins, some natural compounds induce ferroptosis via regulation of the Nrf2 signaling pathway. Withaferin A, a steroidal lactone isolated from the medicinal plant Ashwagandha [*Withania somnifera* (L.) Dunal], and Tiliroside, a flavonoid found in the herbs of *Tribulus terrestris* L., both modulate the Kelch-like ECH-associated protein 1 (Keap1)/Nrf2 pathway in hepatocellular carcinoma. Withaferin A enhances sorafenib sensitivity in sorafenib-resistant hepatocellular carcinoma cells by regulating the Keap1/Nrf2-associated epithelial-to-mesenchymal transition (EMT) and ferroptosis^[44]^. Additionally, tiliroside synergistically combines with sorafenib to inhibit Tank-binding kinase (TBK1) activity, prompting Keap1-mediated Nrf2 ubiquitination and degradation, leading to ferroptosis in hepatocellular carcinoma cells^[45]^. Ginkgetin, a natural biflavonoid isolated from the leaves of *Ginkgo biloba* L., has been found to induce ferroptosis in non-small cell lung cancer cells through decreased expression of xCT and GPX4, decreased GSH/glutathione disulfide (GSSG) ratio, and inactivation of the Nrf2/HMOX1 axis. Interestingly, it can promote DDP-induced anticancer activity, which is also a result of ferroptosis induction^[46]^.

As a key regulator of iron homeostasis, ferritin plays a vital role in storing intracellular free iron and is involved in ferritinophagy, a form of autophagic ferroptosis. In ferritinophagy, the autophagic cargo receptor nuclear receptor coactivator 4 (NCOA4) binds to ferritin heavy chains (FTH1) and delivers it to autophagosomes for degradation and iron release^[47]^. Human carbonyl reductase 1 (CBR1) contributes to gemcitabine resistance in pancreatic cancer. The upregulation of CBR1 induced by gemcitabine inhibits the antitumor effects of the drug. Conversely, reducing CBR1 activity enhances the sensitivity of cancer cells to gemcitabine, thereby improving its therapeutic efficacy. Chrysin, a natural bioflavonoid compound, has been discovered to induce ferritinophagy, thus enhancing gemcitabine sensitivity in pancreatic cancer cells. In Chrysin-treated cells, there is a deregulation of FTH1 and an increase in intracellular free iron levels, followed by the inhibition of CBR1, which is involved in the induction of ferroptosis^[48]^.

The tumor suppressor protein p53 also plays an essential role in ferroptosis in certain cancers. As a diterpenoid extracted from *Torreya nucifera*, kayadiol exhibits anticancer properties through p53-mediated ferroptosis in NK/T lymphoma cells, and it could synergistically combine with L-asparaginase and DDP^[49]^. Artesunate (AST), another derivative of artemisinin, was found to inhibit the growth of sunitinib-resistant renal cell carcinoma cells by both inhibiting cell cycle progression and inducing ferroptosis. Interestingly, the induction of ferroptosis was associated with its inhibitory effect only in renal cell carcinoma cells expressing p53, suggesting that AST induces p53-dependent ferroptosis^[50]^.

## NECROPTOSIS

### Overview of necroptosis

Necroptosis, first described in 2005, is a form of cell death characterized by morphological features similar to necrosis, including a lack of nuclear chromatin, organelle swelling, and cell membrane disruption^[51]^. However, unlike necrosis, which is a passive and non-programmed form of cell death, necroptosis can be regulated by multiple signal transduction pathways^[52]^.

The classical form of necroptotic cell death is mediated by tumor necrosis factor-α (TNF-α). Initially, TNF-α binds to its specific receptor TNF receptor 1 (TNFR1), promoting its trimerization and facilitating the recruitment of several proteins including receptor-interacting protein 1 (RIP1) kinase, TNF-α receptor-associated death domain (TRADD), cellular inhibitor of apoptosis 1 (cIAP1), cellular inhibitor of apoptosis 2 (cIAP2), TNFR-associated factor 2 (TRAF2), and TNFR-associated factor 5 (TRAF5) to form complex I. Within complex I, RIP1 can undergo polyubiquitination by TRAF2, TRAF5, cIAP1, and cIAP2. The ubiquitination status of RIP1 determines whether complex I activates the nuclear factor-κB (NF-κB) pathway to promote cell survival, or triggers cell death^[53]^.

**Figure 2:**
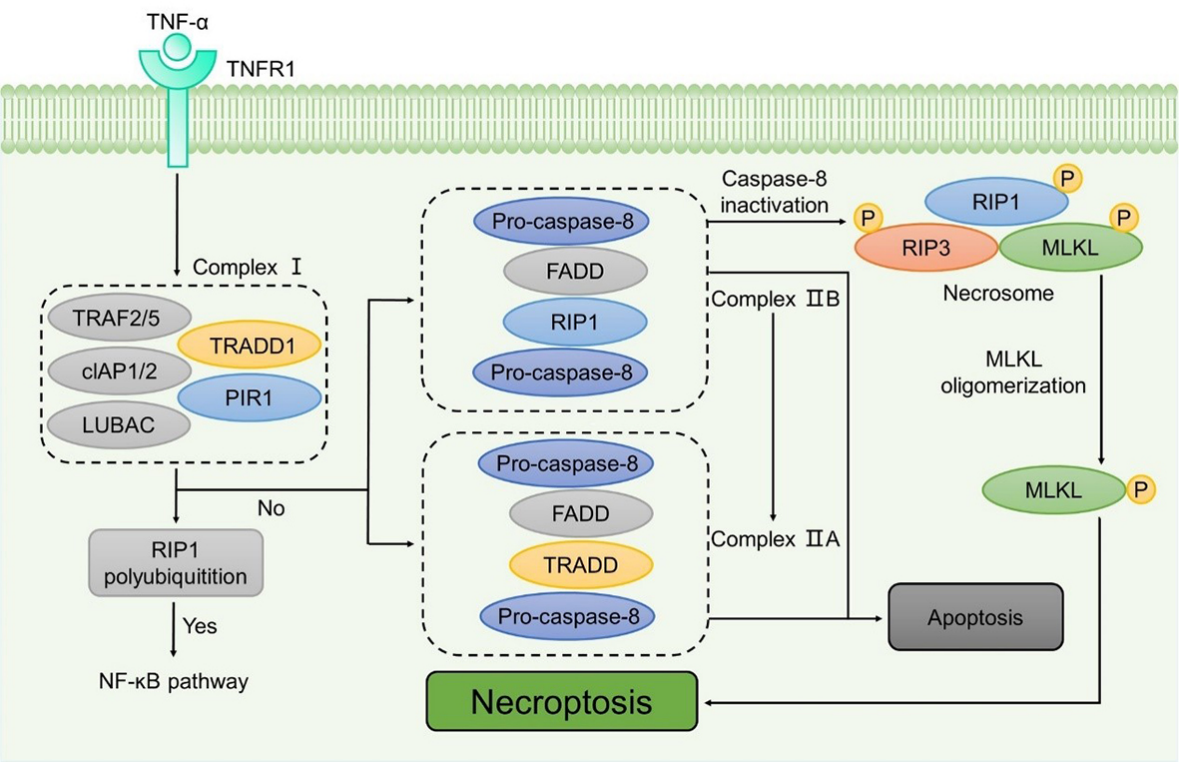
The classical pathway of necroptosis. The classical form of necroptosis begins with the binding of TNF-α to TNFR1, initiating the formation of complex I. If the ubiquitylation of RIP1 within complex I is inhibited, it undergoes a transition into complex IIA and complex IIB. When caspase-8 is inactive, complex IIB recruits RIP3 to form the necrosome. Once RIP3 is phosphorylated, it recruits and phosphorylates MLKL, leading to its oligomerization. The oligomerized MLKL is subsequently translocated to the plasma membrane, resulting in necroptosis. MLKL: Mixed lineage kinase domain-like protein; PIR1: receptor-interacting protein 1; RIP3: receptor-interacting protein 3; TNF: tumor necrosis factor; TNFR: TNF receptor; TRADD: TNF-α receptor-associated death domain TRAF: TNFR-associated factor.

When the ubiquitylation of RIP is impaired, complex I can transform into complex IIa and complex IIb, leading to cell death^[54]^. Complex IIa can trigger caspase-8-dependent apoptosis in the absence of RIP1, whereas complex IIb depends on RIP1 for caspase-8 activation due to its deficiency in TRADD compared to complex IIa^[55]^. Additionally, complex IIa can transform into complex IIb. When the levels of receptor-interacting protein 3 (RIP3) and mixed lineage kinase domain-like protein (MLKL) are sufficiently high and caspase-8 is blocked, complex IIb may develop into a necrosome. Following the phosphorylation of RIP1 and RIP3, two core components of the necrosome, the activated RIP3 can further recruit and phosphorylate MLKL, triggering its oligomerization. The oligomerized MLKL is then translocated to the plasma membrane, increasing its permeability and ultimately leading to necroptotic cell death^[56]^ [Figure 2]. In addition to TNF superfamily receptors, various other types of receptors, such as Toll-like receptors, T-cell receptors, and interferon receptors, also contribute to the activation of necroptosis^[57]^.

### Regulation of necroptosis for anti-drug resistance in cancer

As independent of caspase activation and involving distinct components from apoptotic pathways, necroptosis is an effective mechanism to overcome apoptosis resistance in cancer. However, some necroptotic core components are always lacking in cancer cells, resulting in the evasion of this mechanism. RIP3 is silenced in numerous cancer types, and this silencing is likely ascribed to genomic methylation near the RIP3 transcriptional start site or driven by oncogenes BRAF and AXL^[58]^. Current studies have revealed that demethylation treatment can activate necroptotic pathways by restoring the expression of RIP3. Moreover, upregulating RIPK3 expression can enhance the sensitivity of colon cancer cells to 5-FU and lung cancer cells to DDP through mediating necroptosis^[59,60]^.

Furthermore, the low expression of MLKL appears to be associated with a poor patient prognosis in certain cancers, making it a potential novel potential prognostic biomarker for these cancers^[61]^. In addition, some compounds can induce MLKL-mediated necroptosis without the phosphorylation of RIP1 and RIP3, which presents a very promising prospect for future studies^[62]^.

In addition to modulating necroptosis genes and proteins, increasing glycolytic metabolism may confer resistance to necroptosis in cancer cells under hypoxic conditions. The mechanism involves the suppression of RIP-dependent necroptosis through pyruvate scavenging of mitochondrial superoxide^[63]^. Several studies have suggested that inhibiting glycolysis may be a potential mechanism for necroptosis induction. For instance, selenite-induced necroptosis in prostate cancer resulted from the inhibition of glycolysis through adenosine triphosphate (ATP) depletion and phosphofructokinase activity reduction^[64]^.

### Natural compounds inducing necroptosis for cancer treatment

So far, many natural compounds can induce necroptosis in diverse cancer types, and some can also inhibit drug resistance [Table 2]. As mentioned above, modulating necroptosis core proteins is critical for necroptosis induction. By regulating the RIP1/reactive oxygen species (ROS)-mediated pathway, bufalin, an endogenous cardiotonic steroid, can induce necroptosis in adriamycin-resistant triple-negative breast cancer cell lines^[65]^. Numerous studies have indicated that when 5-FU is in combination with other anticancer agents, its therapeutic efficacy can be effectively enhanced^[66]^. Gambogenic acid is one of the main components of Gamboge which can be used in combination with 5-FU to upregulate necroptosis-related proteins such as RIP1 in lung cancer cells, thereby inducing necroptosis^[67]^. Piperlongumine can also activate RIP1 to produce excessive ROS, triggering necroptosis in DDP-resistant bladder cells^[68]^. Berberine can effectively enhance the antitumor effect of DDP in ovarian cancer by increasing the expression and activation of RIP3 and MLKL, thereby inducing necroptotic cell death^[69]^. In TNF-α induced necroptosis, MLKL is a key downstream component of RIP1 and RIP3. However, it is worth mentioning that tanshinol A, a phenolic compound extracted from *Salvia miltiorrhiza* Bunge, can trigger non-canonical necroptosis mediated by MLKL in lung cancer independently of RIP1 and RIP3^[62]^.

**Table 2:** Natural compounds for anticancer drug resistance by inducing necroptosis

**Compounds**	**Origin**	**Structure**	**Cancer**	**Anti-drug resistant effects**	**Refs**
Berberine	*Coptis chinensis* Franch	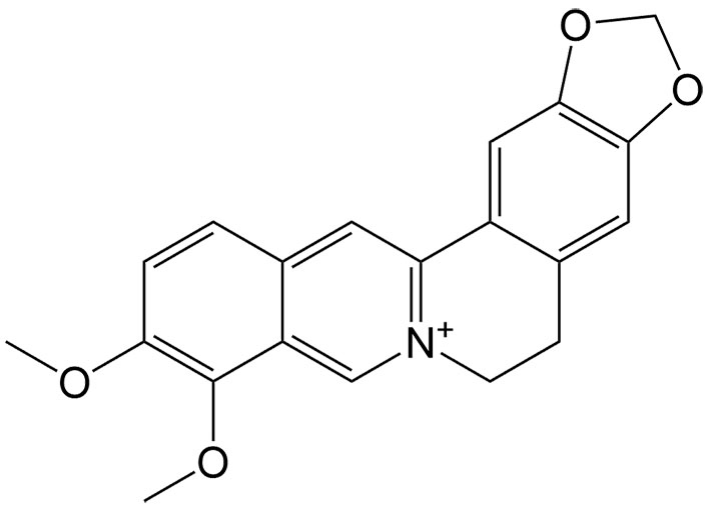	Ovarian cancer	Synergizing with DDP to induce necroptosis by activating the RIP3/MLKL pathway.	[69]
Bufalin	*Bufo bufo gargarizans* Cantor	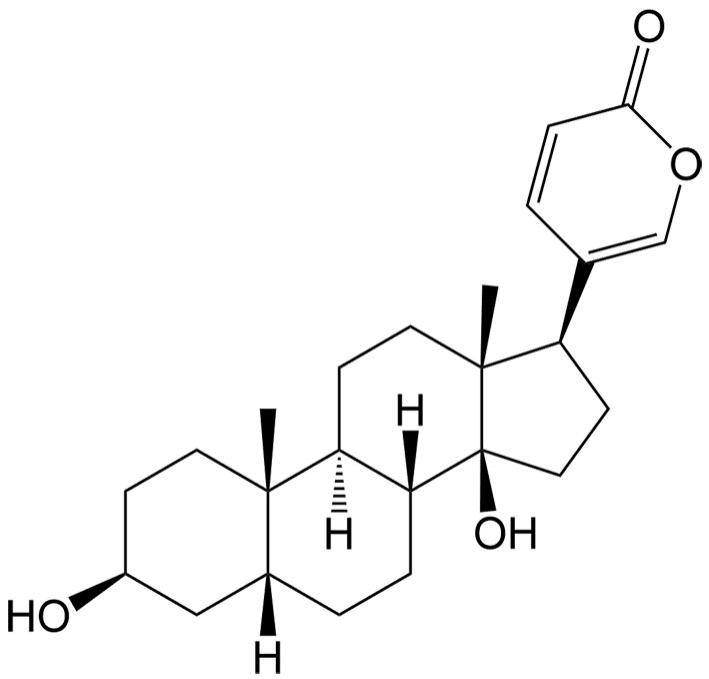	Triple-negative breast cancer	Inducing necroptosis in adriamycin-resistant triple-negative breast cancer through mediating the RIP1/ROS pathway.	[65]
Gambogenic Acid	*Garcinia hanburyi* Hook. f.	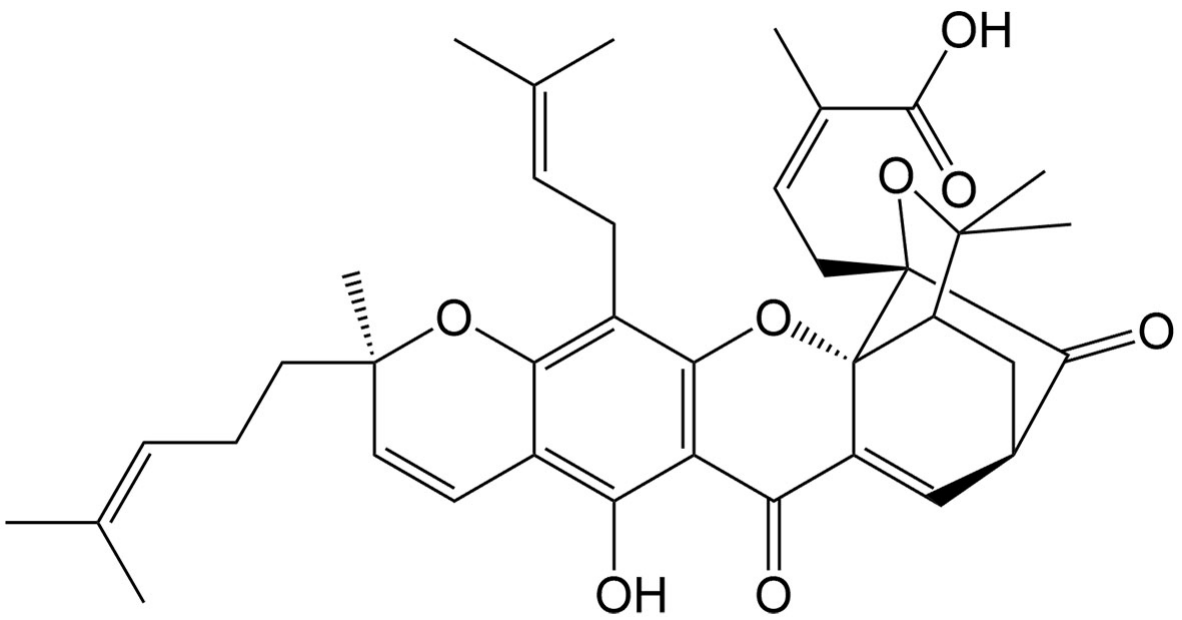	Lung cancer	Synergizing with 5-FU to induce necroptosis by increasing the expression of RIP1.	[67]
Ganoderic acid T	*Ganoderma lucidum* (Leyss.ex Fr.) Karst.	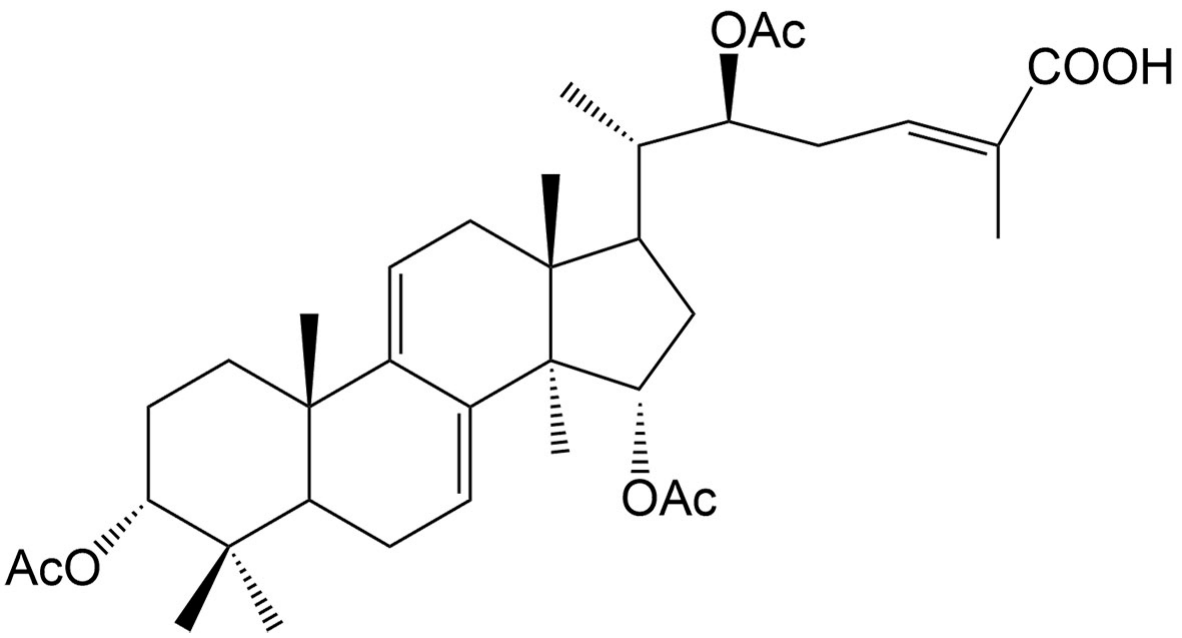	Cervical cancer	Increasing the radiosensitivity of cervical cancer by inducing necroptosis via ROS generation and increased expression of RIP and MLKL.	[71]
Oridonin	*Isodon rubescens* (Hemsley) H. Hara	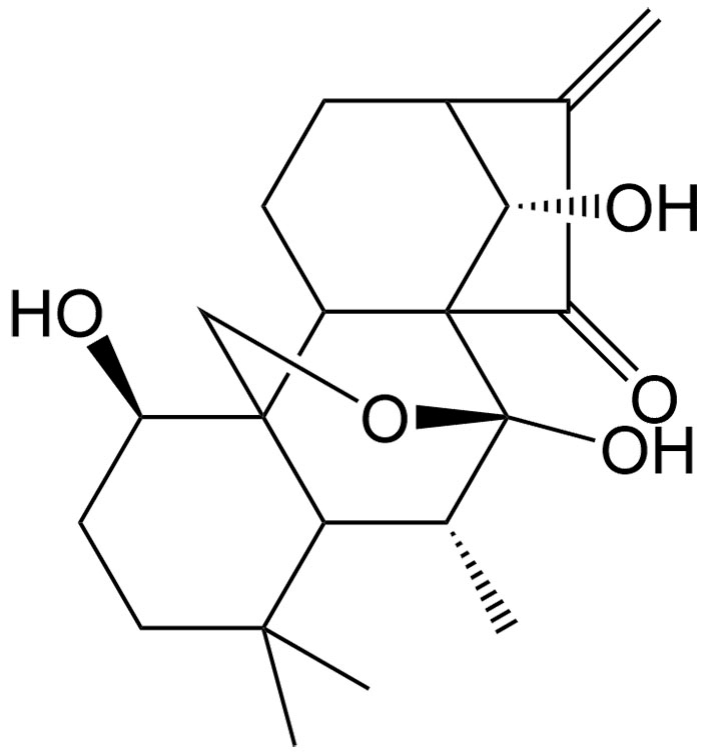	Renal carcinoma	Inducing necroptosis in renal carcinoma to enhance the antitumor effects of 5-FU via ROS generation, GSH depletion, and activation of p38, ERK, and JNK.	[70]

DDP: Cisplatin; ERK: signal-regulated kinase; FU: fluorouracil; GSH: glutathione; JNK: c-Jun N-terminal kinase; MLKL: mixed lineage kinase domain-like protein; RIP: receptor-interacting protein; ROS: reactive oxygen species.

MAPK signaling pathways also play a role in necroptosis induction. For instance, oridonin, a diterpenoid derived from *Isodon rubescens* (Hemsley) H. Hara, has been shown to enhance the cytotoxicity of 5-FU in renal cancer cells by inducing necroptosis. This process is associated with the activation of c-Jun N-terminal kinase (JNK), p38, and extracellular signal-regulated kinase (ERK)^[70]^.

Moreover, certain studies have demonstrated that the inhibition of caspases may cause a switch from apoptosis to necroptosis in specific cancer types. Ganoderic acid T (GAT) is a triterpene of *Garcinia hanburyi* Hook. f., inducing both necroptosis and apoptosis in cervical cancer cells. Interestingly, the percentage ratio of necroptosis is increased following the increase of GAT concentration, as GAT can reduce the matrix metalloproteinase (MMP) and ATP levels and caspase-8 expression under radiation conditions^[71]^.

Notably, glycolysis suppression has also emerged as an effective mechanism for necroptosis induction by natural compounds. Docetaxel is indeed a valuable chemotherapeutic agent utilized in the treatment of prostate cancer, primarily by inducing cell death^[72]^. However, similar to DDP, the effectiveness of docetaxel-induced apoptosis can also be hindered through various pathways, including the p38/p53/p21 pathway, USP33-DUSP1-JNK pathway, and PI3K/Akt/NF-κB pathway^[73-75]^. Therefore, several studies are currently focused on identifying alternative cell death pathways, such as necroptosis, that can be induced in docetaxel-resistant cancer cells as well. Shikonin has been extensively studied as a natural necroptosis inducer in various cancer types, and it has been demonstrated to overcome drug resistance to docetaxel in prostate cancer and DDP in bladder cancer^[76,77]^. Furthermore, shikonin can induce glycolysis suppression in glioma cells, which is closely associated with the accumulation of intracellular H_2_O_2_ triggered by the activation of RIP1 and RIP3^[78]^.

## PYROPTOSIS

### Overview of pyroptosis

Pyroptosis, a pro-inflammatory programmed cell death, was originally termed by Cookson *et al.* in 2001^[79]^. The term derives from the Greek roots “pyro”, which relates to fire or fever, and “ptosis”, denoting a falling, reflecting its nature. Pyroptosis shares certain characteristics with apoptosis, such as DNA fragmentation, nuclear condensation, and caspase dependence. However, cells undergoing pyroptosis differ in that they retain intact nuclei and exhibit pore formation in the plasma membrane^[80]^. An increasing understanding of pyroptosis has revealed that this type of cell death can be divided into classical and non-classical pathways.

The classical pyroptotic pathway is mediated by inflammasome assembly, which consists of pattern recognition receptors (PRRs), apoptosis-related speck-like protein (ASC), and pro-caspase-1^[81]^. PRRs, functioning as inflammasome sensors, recognize pathogen-associated molecular patterns (PAMPs) and damage-associated molecular patterns (DAMPs)^[82]^. Subsequently, they recruit the bridging protein ASC, which contains a pyrin domain (PYD) and a caspase activation and recruitment domain (CARD), through specific PYD-PYD interactions. After being recruited, ASC can interact with and activate pro-caspase-1 via CARD-CARD interactions^[83]^. Some PRRs containing CARD can also directly bind to pro-caspase-1, forming inflammasomes without the participation of ASC^[84]^. Activated caspase-1 facilitates the maturation of inflammatory cytokines interleukin (IL)-1β and IL-18, as well as cleaves the pore-forming protein gasdermin D (GSDMD) to produce its N-terminal fragment (N-GSDMD)^[85]^. N-GSDMDs then translocate to the plasma membrane and form pores, promoting the release of mature IL-1β and IL-18. As the number of N-GSDMD pores increases, cells swell and rupture, resulting in pyroptotic cell death^[86]^.

**Figure 3:**
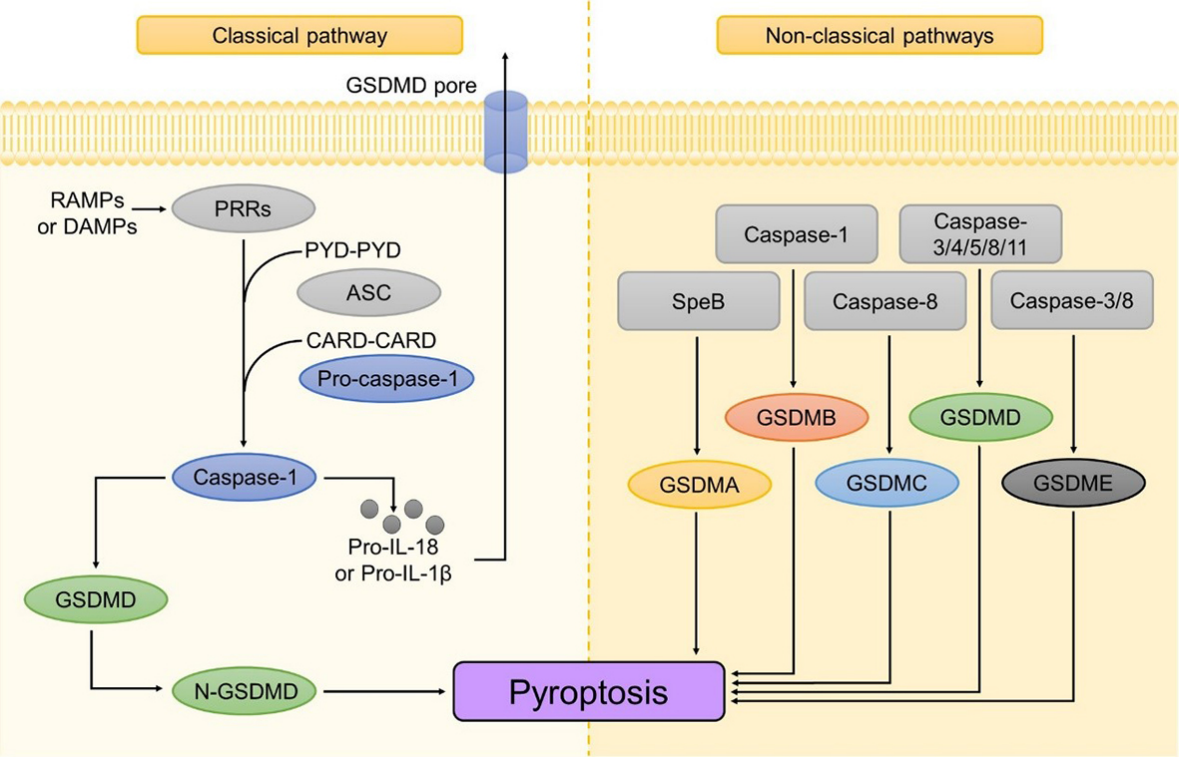
In the classical pathway, PRRs recognize PAMPs and DAMPs, initiating the recruitment of ASC and pro-caspase-1. The activation of caspase-1 results in cleavage of GSDMD, forming GSDMD pores that ultimately trigger pyroptosis. Furthermore, caspase-1 also results in the maturation of IL-1β and IL-18, which are eventually released from the GSDMD pores. Alternatively, in the non-classical pathway, pyroptosis can be initiated by other members of the GSDM family. CARD: Caspase activation and recruitment domain; DAMPs: damage-associated molecular patterns; GSDMD: gasdermin D; IL: interleukin; PAMPs: pathogen-associated molecular patterns; PRR: pattern recognition receptor; PYD: pyrin domain.

In non-classical pathways, caspase-3/8 can also trigger pyroptosis by activating GSDMD or GSDME^[87,88]^. Additionally, caspase-8 can induce GSDMC-dependent pyroptosis as well^[89]^. The cleavage of GSDMD can also be mediated by caspase-4/5/11, which recognizes intracellular LPS to activate the non-canonical inflammasome^[90]^. Notably, GSDMB can not only be cleaved by caspase-1 to directly initiate pyroptosis, but also enhance caspase-4 activity to promote this cell death mechanism^[91]^. Recently, the cleavage of GSDMA has been found to be catalyzed by streptococcal pyrogenic exotoxin B (SpeB), a cysteine protease secreted by group A Streptococcus. This finding demonstrates that GSDMA can also play a role in pyroptosis by releasing the cleaved N-terminal fragments, which can bind to and disrupt specific acidic lipid-containing membranes^[92]^ [Figure 3].

### Induction of pyroptosis for anti-drug resistance in cancer

In cancer treatment, the induction of pyroptosis is increasingly recognized as a promising strategy for overcoming drug resistance. NOD-like receptor family pyrin domain-containing 3 (NLRP3), a crucial inflammasome sensor in the NLR family, is recognized as a downstream target of multiple microRNAs (miRNAs) associated with cancer drug resistance. For example, by downregulating the expression of miR-556-5p in non-small cell lung cancer, NLRP3 inflammasome-mediated pyroptosis can be triggered, thereby enhancing DDP sensitivity^[93]^.

The pore-forming proteins GSDMD and GSDME, extensively studied in the GSDM family, are potential targets for combating drug resistance and contributing to the treatment and prognosis of various cancers. Some chemotherapeutic agents have been demonstrated to exert antitumor activity when used alone or in combination to induce GSDMD-dependent pyroptosis, enhancing the efficacy of chemotherapy. For instance, the co-administration of paclitaxel and ruthenium complexes can induce cell death in paclitaxel-resistant cervical cancer cells by mediating Caspase-1/GSDMD-dependent pyroptosis^[94]^. GSDME-mediated pyroptosis has been observed to improve the sensitivity of various drugs across different cancer types, including increasing DDP sensitivity in esophageal squamous cell carcinoma cells and oxaliplatin sensitivity in colon cancer cells^[95]^. Additionally, it can alleviate the side effects of DDP in patients with oral squamous cell carcinoma.

In recent years, programmed death-ligand 1 (PD-L1), known as an immune checkpoint, has emerged as a research hotspot in tumor immunotherapy. However, PD-L1 has an additional role as a non-immune checkpoint by regulating the non-classical pyroptosis pathway mediated by GSDMC/caspase-8^[96]^. In this way, a variety of antibiotics can induce pyroptosis in cancer cells, indicating that it could be a novel strategy to combat antibiotic resistance in chemotherapy.

### Natural compounds inducing pyroptosis for cancer treatment

Nowadays, the caspase-1/GSDMD and caspase-3/GSDME pathways have attracted significant attention in pyroptosis induction, and many natural compounds have been found to activate these pathways in cancers [Table 3]. Wedelolactone, an ingredient of *Eclipta prostrata* (L.) L., can simultaneously activate these two pathways by strongly increasing the activation of caspase-1, caspase-3, GSDME and GSDMD in retinoblastoma cells^[97]^. Ophiopogonin B, derived from *Dioscorea bulbifera* L., can induce caspase-1/GSDMD-dependent pyroptosis in lung cancer cells, especially exhibiting a more significant suppression of growth in DDP-resistant cancer cells^[98]^. As a pentacyclic triterpene compound of lupine, betulinic acid can induce caspase-1-dependent pyroptosis, thereby enhancing chemosensitivity to DDP in esophageal cancer cells^[99]^.

**Table 3:** Natural compounds for anticancer drug resistance by inducing pyroptosis

**Compounds**	**Origin**	**Structure**	**Cancer**	**Anti-drug resistant effects**	**Refs**
Betulinic acid	*Betula platyphylla* Suk.	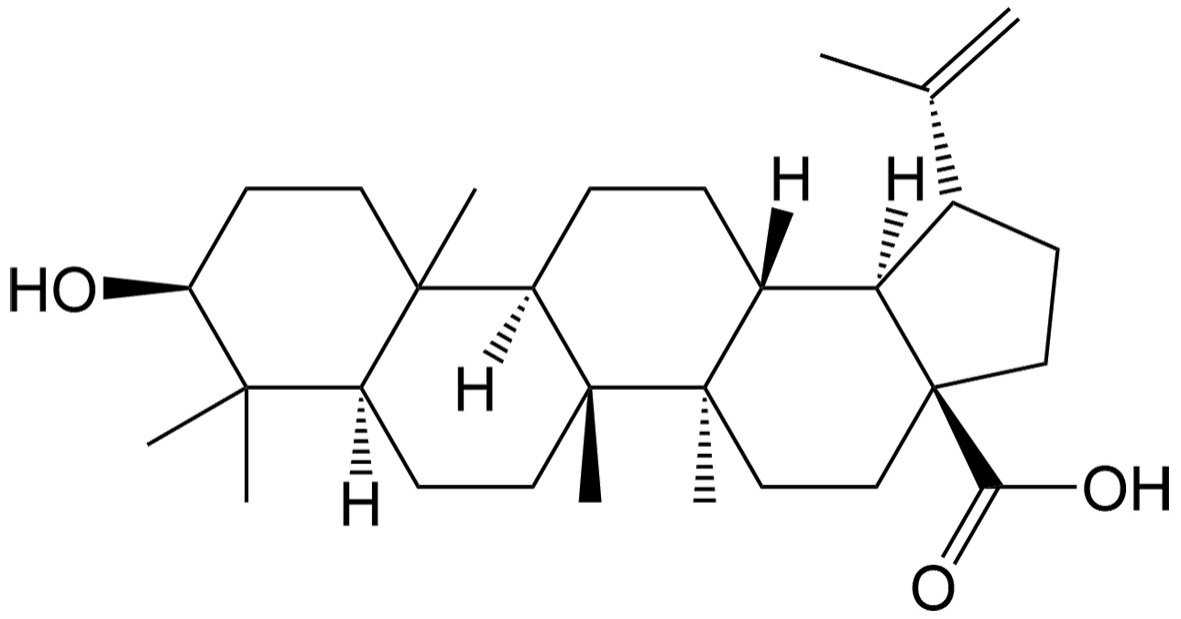	Esophageal cancer	Enhancing the antitumor effects of DDP by inducing pyroptosis via increasing the levels of ASC and caspase-1 and decreasing the levels of Ki67, PCNA, SOX2, and OCT4.	[99]
Diosbulbin B	*Dioscorea bulbifera* L.	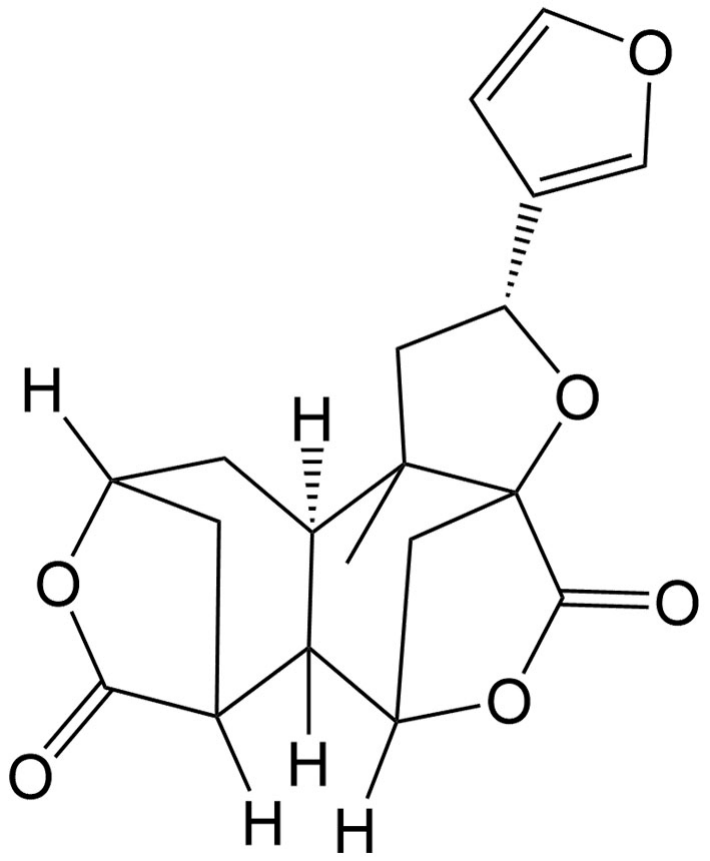	Gastric cancer	Increasing DDP-sensitivity in gastric cancer by inducing pyroptosis via regulating the PD-L1/NLRP3 pathway.	[106]
Ophiopogonin B	*Ophiopogon japonicus* (L. f.) Ker-Gawl.	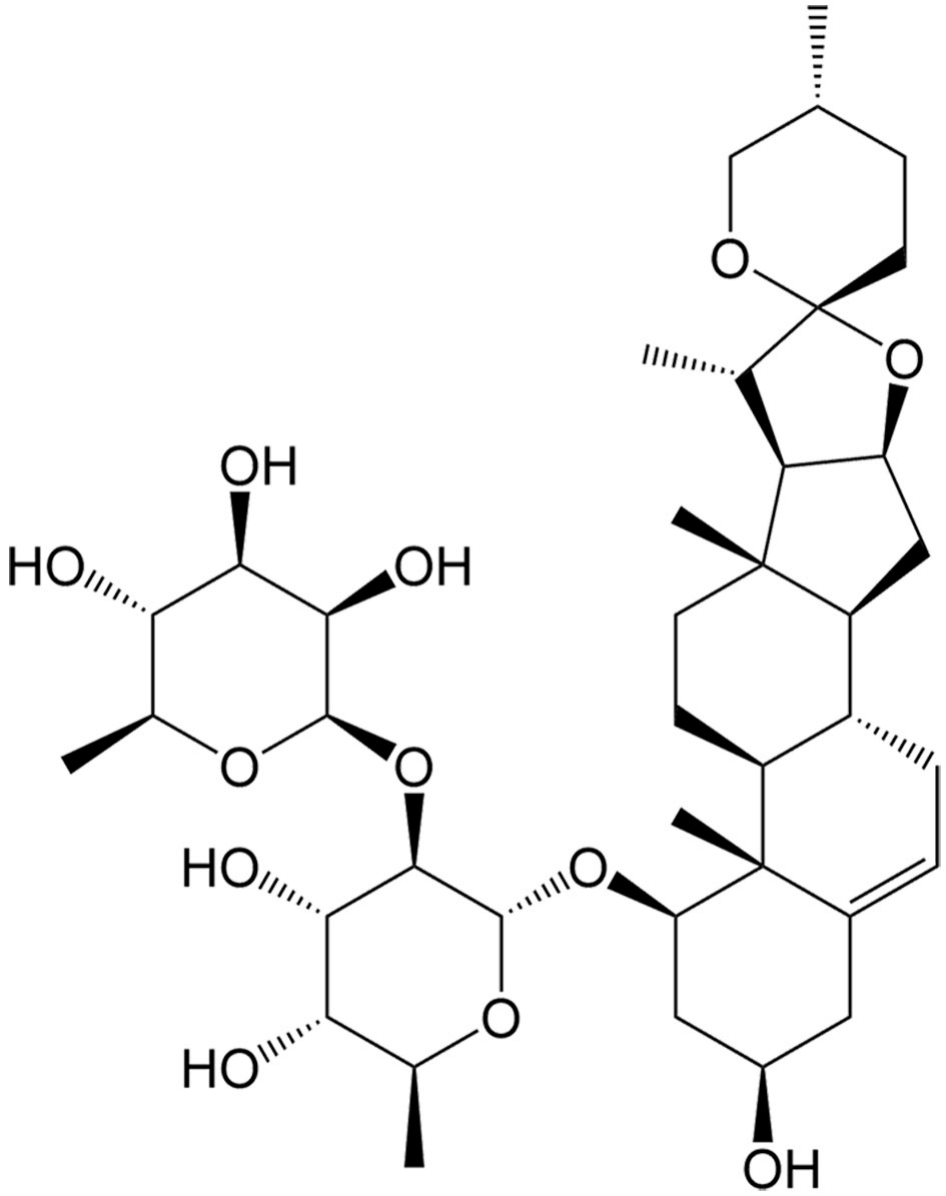	Lung cancer	Increasing DDP-sensitivity in gastric cancer by inducing pyroptosis via regulating the caspase-1/GSDMD pathway.	[98]

ASC: Apoptosis-related speck-like protein; DDP: cisplatin; GSDMD: gasdermin D; NLRP3: NOD-like receptor family pyrin domain-containing 3; OCT4: octamer-binding transcription factor-4; PCNA: proliferating cell nuclear antigen; PD-L1: programmed death-ligand 1; SOX2: SRY-related high mobility group box protein-2.

By activating the caspase-3-dependent pathway, natural compounds such as curcumin, dihydroartemisinin, and germacrone can induce pyroptosis in different cancers^[100-102]^. Some studies have reported that the activation of caspase-9 is also involved in caspase-3-mediated pyroptosis. For example, alantolactone, a terpenoid of *Inula helenium* L., can promote the cleavage of caspase-9 and caspase-3 to induce GSDME-mediated pyroptosis in anaplastic thyroid cancer^[103]^. Some chemotherapeutic agents, such as DDP, 5-FU, and carboplatin, have been demonstrated to combat cancer drug resistance by inducing GSDME-dependent pyroptosis, while whether natural compounds can also suppress drug resistance through this pathway needs further investigation^[95,104,105]^.

In addition, by downregulating PD-L1 to activate NLRP3-mediated pyroptosis, Diosbulbin B extracted from *Dioscorea bulbifera* L. can sensitize DDP-resistant gastric cancer cells to DDP^[106]^. At present, there are few reports on whether natural compounds induce pyroptosis via the regulation of other GSDM family proteins. This is partly because of their unclear functions in initiating pyroptotic cell death. Further elucidation of how the other members of the GSDM family contribute to pyroptosis induction in cancers may provide new insights for the search for natural compounds with anticancer and anti-drug resistance activities.

## PARAPTOSIS

### Overview of paraptosis

Paraptosis was first introduced as a form of programmed cell death by Sperandio *et al.* in 2000^[107]^. It derives from “para”, meaning “next to” or “related to”, and “apoptosis”, suggesting that it is distinct from apoptosis. The main morphological features of paraptosis include cytoplasmic vacuolization, swelling of the endoplasmic reticulum and/or mitochondria, and the absence of nuclear fragmentation or apoptotic body formation. In this paradigm, caspases are not activated, and thus, cells undergoing paraptosis are resistant to caspase inhibitors^[108]^.

**Figure 4:**
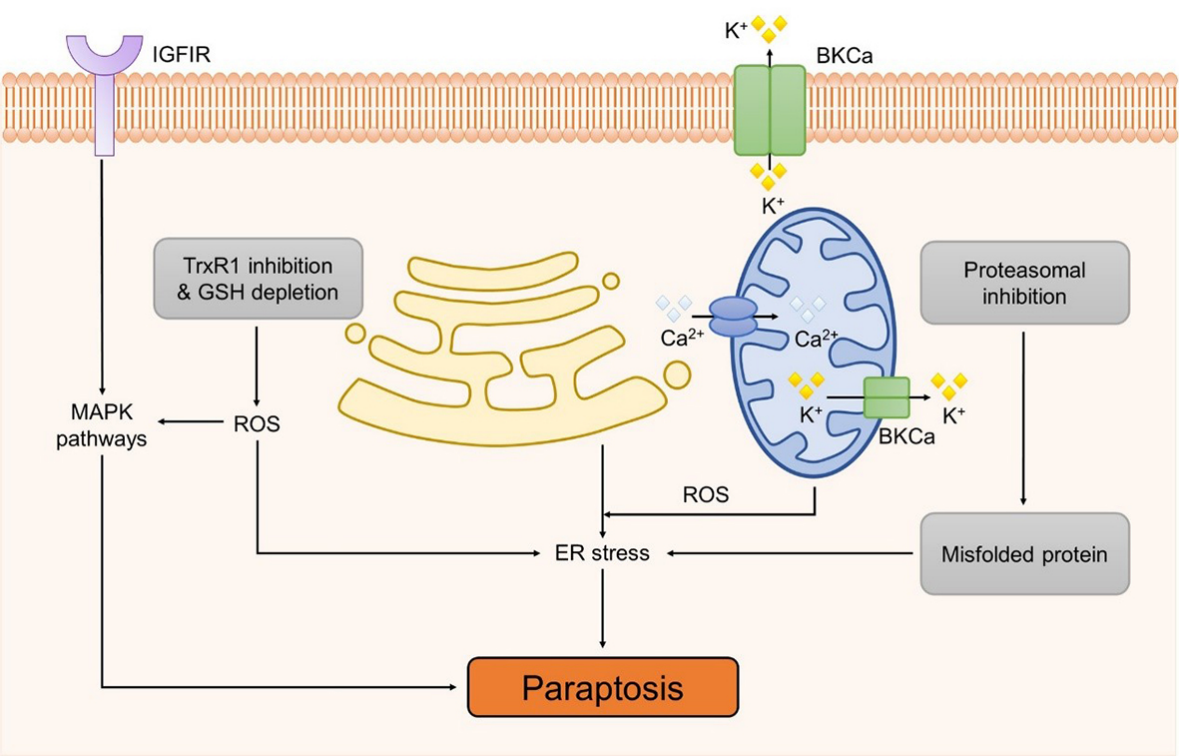
The mechanism of Paraptosis. Paraptosis was initially discovered to be induced by IGFIR and mediated through the MAPK pathways. Furthermore, it is intricately linked to various factors, including TrxR1 inhibition, GSH depletion, intracellular Ca^2+^ homeostasis, activation of BKCa channels, and proteasome inhibition. BKCa: Ca^2+^-activated K^+^; ER: endoplasmic reticulum; GSH: glutathione; IGFIR: insulin-like growth factor-I receptor; MAPK: MAP kinase; ROS: reactive oxygen species.

The MAPK pathways play a critical role in paraptosis, and research focusing on the ERK, JNK, and p38 pathways is particularly extensive. Notably, the protein AIP1/Alix was described as the first specific inhibitor of paraptosis, capable of restraining the insulin-like growth factor-I receptor (IGFIR) induced paraptotic process mediated by the MAPK/ERK and JNK pathways^[109]^. Furthermore, TrxR1 inhibition and GSH depletion have been observed to potentially activate the MAPK pathways by triggering the accumulation of cellular ROS^[110]^. Paraptosis induction is also related to the homeostasis of intracellular Ca^2+^, which is mainly regulated by the endoplasmic reticulum and mitochondria. Intracellular Ca^2+^ can be released from the endoplasmic reticulum into mitochondria when paraptosis is initiated, resulting in the endoplasmic reticulum and mitochondrial dilation and ultimately leading to cell death. The voltage- and Ca^2+^-activated K^+^ (BKCa) channels are widely expressed in body and have the ability to link changes in intracellular calcium to outward hyperpolarizing potassium currents. The activation of these channels will disrupt the osmotic balance, initiating cell swelling and vacuolization^[111]^. Additionally, proteasome inhibition may also promote paraptosis by inducing endoplasmic reticulum (ER) stress^[112]^ [Figure 4].

### Paraptosis potential applications in cancer drug resistance

Paraptosis induction is a potential strategy for developing non-genetically modified tumor vaccines. In rat T9 glioma cells, the activation of BKCa channels promotes the overexpression of heat shock proteins and the translocation of HMGB1 from the nuclear region to the periphery, stimulating immune responses and initiating paraptosis. Rats injected with paraptotic T9 glioma cells, which are killed by prolonged BKCa channel activation, can develop specific immunity to T9 cells. This suggests the potential of using these treated cells as a functionally killed vaccine^[113]^.

Due to its unique molecular mechanism, paraptosis induction also contributes to enhancing the activity of proteasome inhibitors in cancer cells. Although many proteasome inhibitors have shown antitumor activities, their clinical efficacy is unsatisfactory as their effectiveness can be compromised by both primary and secondary resistance mechanisms. Therefore, combination therapy can be seen as an effective strategy to address proteasome inhibitor resistance. For instance, by triggering paraptotic cell death, bortezomib (Btz), a 20S core particle inhibitor of the proteasome, can be combined with loperamide, an antidiarrheal agent, to enhance Btz sensitivity and reduce its side effects, effectively combating the colon cancer^[114]^.

### Natural compounds inducing paraptosis for cancer treatment

Currently, a number of natural compounds have shown potential in cancer treatment by inducing paraptosis [Table 4], with many of them modulating the MAPK signaling pathways to trigger ER stress. One such example is Paris Saponin II, derived from *Paris polyphylla* Smith, which effectively induces paraptosis by activating the JNK pathway and augmenting ER stress^[115]^. Moreover, the activation of this paraptosis-associated pathway enhances the sensitivity of DDP in lung cancer. Jolkinolide B, an active abietane ent-diterpenoid, is also a noteworthy compound that induces paraptosis in both sensitive and DDP-resistant bladder cancer cells by activating the ERK pathway and enhancing ER stress^[110]^. Additionally, Chalcomoracin, isolated from *Morus alba* L., has been discovered to enhance the sensitivity of non-small cell lung cancer to radiation by augmenting ER stress^[116]^. Elaiophylin is a natural antibiotic derived from *Streptomyces melanosporus* that can also induce paraptosis through the hyperactivation of the MAPK pathway. This compound demonstrates notable efficacy in eliminating ovarian cancer cells that are resistant to multiple drugs, including platinum, taxane, and poly (ADP-ribose) polymerase inhibitor (PARPi)^[117]^.

**Table 4:** Natural compounds for anticancer drug resistance by inducing paraptosis

**Compounds**	**Origin**	**Structure**	**Cancer**	**Anti-drug resistant effects**	**Refs**
Chalcomoracin	*Morus alba* L.	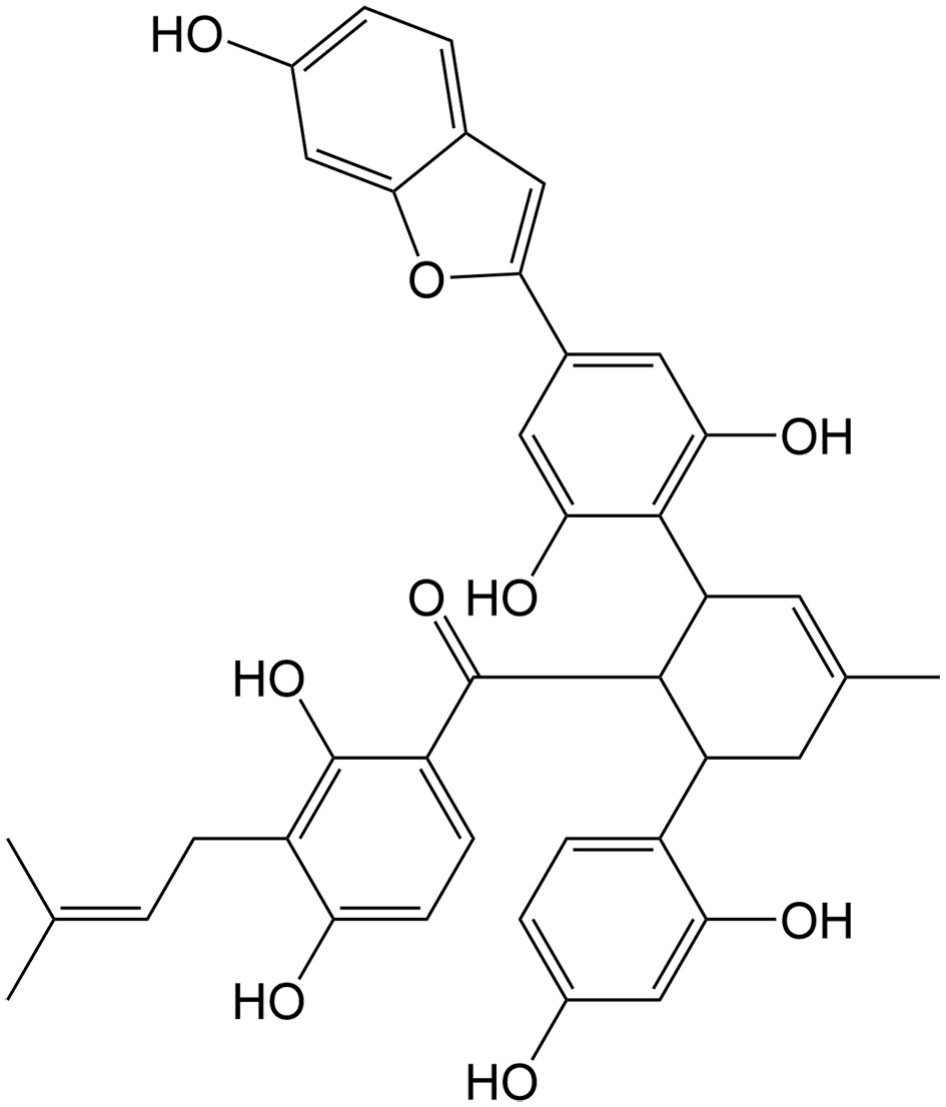	Non-small cell lung cancer	Increasing the radiosensitivity of non-small cell lung cancer by inducing ER stress-mediated paraptosis via activation of the MAPK pathway.	[116]
Elaiophylin	*Streptomyces hygroscopicus*	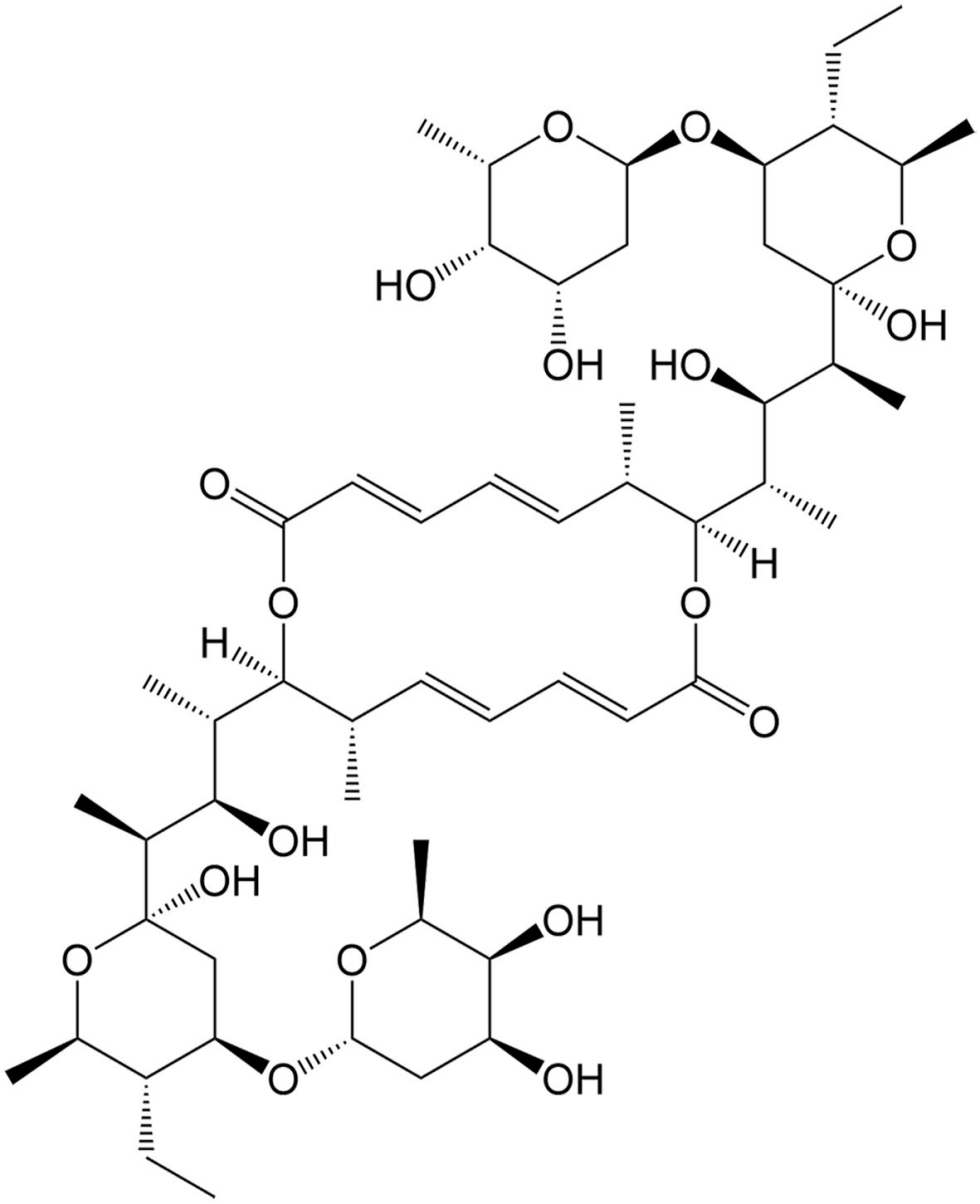	Ovarian cancer	Inducing paraptosis to overcome platinum, taxane, and PARPi resistance in ovarian cancer by regulating the SHP2/SOS1/MAPK pathway.	[117]
Jolkinolide B	*Euphorbia fischeriana* Steud	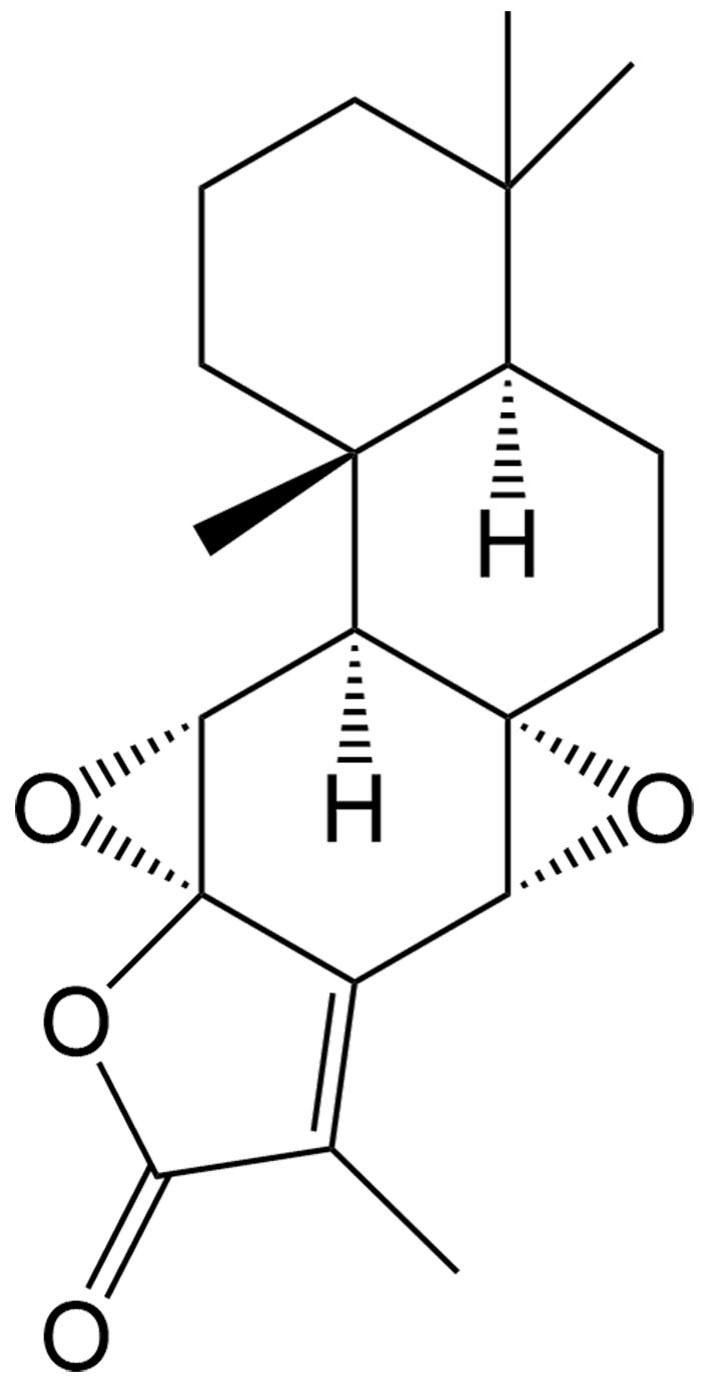	Bladder cancer	Inducing ROS-mediated paraptosis to suppress the growth of DDP-resistant bladder cancer by targeting thioredoxin and glutathione systems.	[110,131]
Paris saponin II	*Paris polyphylla* Smith	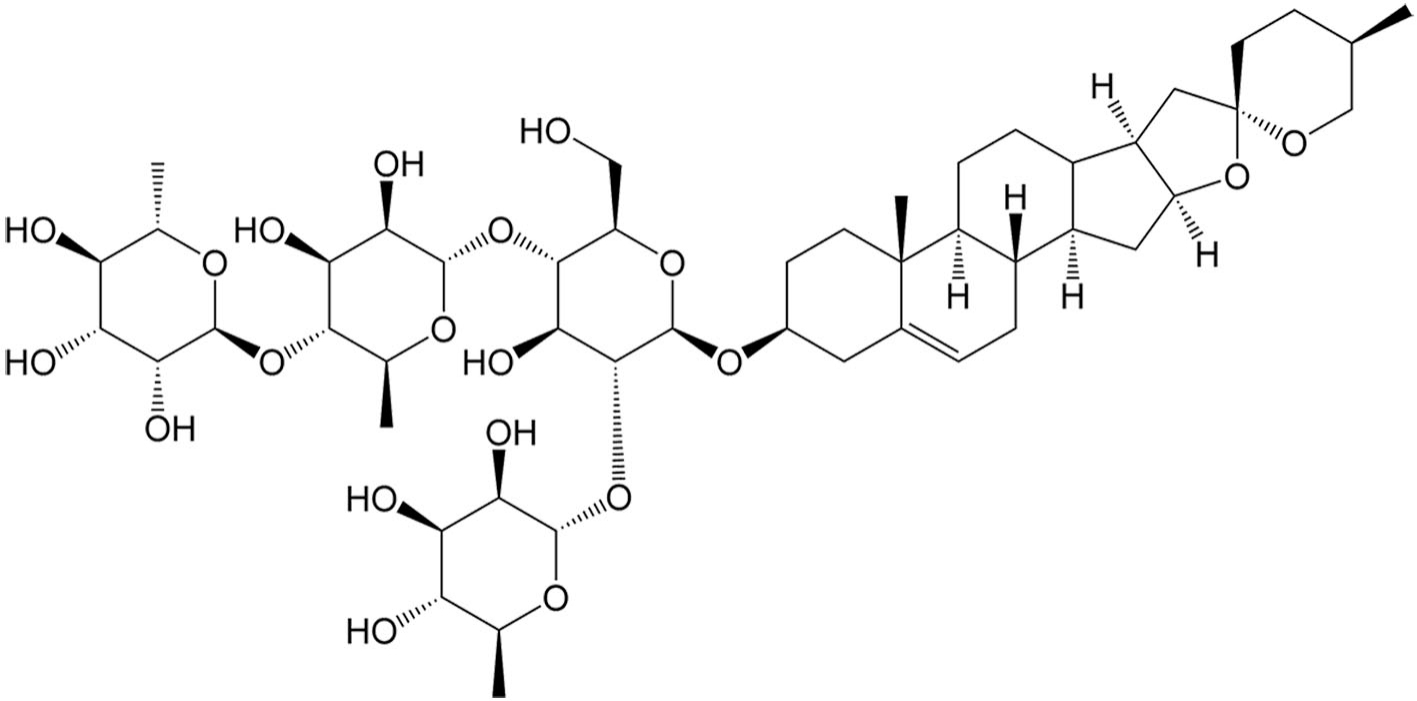	Non-small cell lung cancer	Enhancing the antitumor effects of DDP by regulating the JNK pathway.	[115]

DDP: Cisplatin; ER: endoplasmic reticulum; JNK: c-Jun N-terminal kinase; MAPK: MAP kinase; PARPi: poly (ADP-ribose) polymerase inhibitor; ROS: reactive oxygen species; SHP2: src homology 2 domain-containing tyrosine phosphatase 2; SOS1: son of sevenless homolog 1.

Some natural compounds such as curcumin, morusin, and ophiobolin A can also induce paraptosis by affecting ion homeostasis. Curcumin can induce paraptosis in epithelial ovarian cancer mainly through mitochondrial Ca^2+^ overload, which subsequently contributes to mitochondrial swelling and dysfunction^[118]^. Similarly, morusin, a prenylflavonoid, can also induce paraptosis in breast cancer by triggering mitochondrial Ca^2+^ overload^[119]^. In glioblastoma cells, paraptosis induced by ophiobolin A, a sesterterpenoid phytotoxin from the genus Bipolaris, is linked to K^+^ homeostasis imbalance, primarily caused by the blockage of BKCa channels^[120]^.

Furthermore, natural compounds can induce paraptosis by disrupting sulfhydryl homeostasis and suppressing proteasome functions, both of which can be significantly inhibited by thiol antioxidants. In this way, plumbagin, extracted from *Plumbago zeylanica* L., can induce paraptotic cell death in different cancer types^[121]^. Paraptosis can also be triggered in a p53-dependent manner. For instance, Ginsenoside Rh2, which is a bioactive product in *Panax ginseng* C. A. Meyer, can induce paraptosis in colorectal cancer via activating the p53 pathway as well as the NF-κB survival pathway^[122]^.

## CONCLUSION

Numerous natural compounds possess the ability to elicit anticancer and anti-chemoresistance effects by triggering non-apoptotic cell death mechanisms such as ferroptosis, necroptosis, pyroptosis, and paraptosis. It is noteworthy that while these forms of cell death have unique regulators, some common pathways can also govern them. This implies that multiple cell death pathways may occur concurrently and be subject to simultaneous regulation. In 2019, Malireddi *et al.* introduced PANoptosis, an innovative form of cell death that amalgamates essential features of pyroptosis, apoptosis, and necroptosis yet defies a straightforward classification under any one of these categories^[123]^. This discovery underscores the co-regulation and interplay among these cell death pathways, suggesting that drugs may possess multifaceted regulatory effects by modulating master factors within these pathways. Beyond the four aforementioned types of cell death, some other novel cell death mechanisms have surfaced in recent years, including parthanatos, disulfidptosis, and cuproptosis^[124-126]^. These revelations open up new avenues for identifying targets in cancer treatment and provide additional strategies for combating cancer drug resistance. As such, ongoing exploration is essential to ascertain whether natural compounds can elicit anticancer effects through these emerging forms of cell death.

## DECLARATIONS

### Copyright

© The Author(s) 2023.
